# Skin-reducing mastectomy and immediate breast reconstruction in patients with macromastia

**DOI:** 10.1186/s13046-015-0227-5

**Published:** 2015-10-14

**Authors:** Roy De Vita, Marcello Pozzi, Giovanni Zoccali, Maurizio Costantini, Pierpaolo Gullo, Ernesto Maria Buccheri, Antonio Varanese

**Affiliations:** Department of Plastic and Reconstructive Surgery, IFO - “Regina Elena” National Cancer Institute, Via Elio Chianesi 53, Rome, Italy; Department of Life, Health and Environmental Sciences, Plastic Reconstructive and Aesthetic Surgery Section, L’Aquila University, L’Aquila, Italy

**Keywords:** Skin reducing mastectomy, Immediate breast reconstruction, ADM, SRM, Breast implant, Skin sparing mastectomy

## Abstract

**Introduction:**

In women with macromastia, a type IV skin sparing mastectomy is often required to achieve an aesthetically pleasing reconstruction. The introduction of “skin-reducing mastectomy”, which inserts a permanent prosthetic device into a large pouch made by the pectoralis major muscle and an inferior pedicle dermal flap, allows the surgeon to achieve a safe oncologic result plus a cosmetically satisfying reconstruction.

**Objective:**

We report here our experience with the skin-reducing mastectomy with the aim of emphasizing the problems associated with the technique.

**Materials and method:**

A study was conduced from April 2009 to November 2012, 74 patients with breast cancer were selected then received a skin-reducing mastectomy. The enrolled women satisfied the criteria of Nava. Statistical analysis was conduced to estimate the significance of the outcome results and complication rate.

**Result:**

A total of 88 SRMs were performed and the outcomes were as follows: excellent in 34 patients, good in 21, and poor in 8. We recorded 25 % of adverse events and statistic helped us to identify patient related factors whose can increase the complications onset.

**Conclusion:**

Skin-reducing mastectomy is an indispensable procedure to treat cancer in large and pendulous breasts.

The correct patient selection is mandatory to reduce the not negligible complication risk. Skin reducing mastectomy when is well conduced allow to obtain good results with patient satisfaction but, like other breast-conserving surgeries, in some case is not the ultimate solution, because it may require some revisions to maintain the optimum results.

To day it be consider relatively safe in selected patient and the gold standard in macormastia immediate reconstruction.

## Introduction

Loosing a breast to cancer has significant negative psychological and sexual consequences [1], and one aim of patient care is to ameliorate these problems. Therefore, the radical mastectomy has become less common and has been replaced by wide local excision (breast-conserving therapy) or mastectomy followed by reconstruction [2, 3].

Skin-sparing mastectomy (SSM) was first described in 1991 by Toth et al. [4] as an attempt to maximize skin preservation in order to provide a more cosmetically pleasing result after reconstruction. SSM is now considered an oncologically safe surgical procedure [5–9].

Carlson et al. [10] described 4 types of SSMs, which were based on the type of incision and the amount of excised skin. SSM types I to III are intended for small breasts with a small degree of ptosis, and are carried out using a periareolar approach. The type IV SSM is intended for heavy, pendulous breasts that require large reduction of the skin envelope plus reduction or mastopexy of the contralateral breast [11]. This surgical procedure has 2 main limitations: the first is the risk for ischemic necrosis of the two long and thin superior flaps that close down to the inframammary fold, which may lead to complications in healing of the inverted T-scar, such as superficial epidermolysis, wound dehiscence, and exposure of the implant. The second limitation is excessive upper pole fullness caused by a permanent implant in the lower pole of the reconstructed breast that lacks projection [11–13].

To increase the coverage of the lower-pole implant and reduce the risk of device infection due to wound dehiscence or skin necrosis, a technique was developed that used the skin of the lower breast to create an inferior dermal pedicle flap sutured to the inferior border of the pectoralis major muscle, thereby creating a musculodermal pouch for a definitive implant [14]. This procedure was described by Bostwick and was introduced primarily for cosmetic surgery and cancer prophylaxis [15]. Hammond subsequently expanded its use to breast cancer, although purely for a two-stage reconstruction that used an expander for the first stage and a definitive implant for the second stage [16]. In 2006, Nava et al. reviewed this relatively unknown technique naming it as “skin-reducing mastectomy” [11].

We report herein our experience performing the skin-reducing mastectomy at the Regina Elena National Cancer Institute in Rome, Italy, with the aim of emphasizing the problems associated with the technique.

## Materials and methods

### Patient selection

This was a retrospective study from April 2009 to November 2012 of 74 patients selected by our department; the local ethics committee approved this study.

Patient data, including age, body mass index (BMI, patients with BMI > 35 were excluded), smoking status, and comorbid conditions were recorded. Indications for surgery, including type of cancer, breast size, and grade of ptosis were also recorded. The patients were enrolled in this study based on the criteria of Nava [11], which included patients with pendulous breasts and an areola-to inframammary fold distance greater than 8 cm and suprasternal notch-to-nipple distance greater than 25 cm. Table 1 summarizes the characteristics of our patients.

**Table 1:** Patient description

Demographic characteristic	
Average age (range)	52 years (26 – 67)
Average BMI (range)	27.6 (21 – 34)
Anatomical characteristic	
Average breast width (range)	15.8 cm (12 – 17.6)
Average nipple to Inframammary fold distance (range)	11.7 cm (8 – 17.5)
Average nipple to sterna nock distance (range)	27.9 cm (25 – 36.8)

None of the study patients had serious comorbidities that contraindicated surgery or had previously undergone to breast surgery. Eight patients had diabetes (16.2 %) and 16 patients (21.6) were smokers. All patients who were referred to our department needing postoperative radiotherapy due to clinical positivity of axilla were not enrolled to receive a prosthetic implantation but were scheduled for autologous tissue reconstruction. All the study patients fulfilled the oncological indications for undergoing a skin-sparing mastectomy. Before surgery they consented to the procedure, the permission to be enrolled in the study, to take photos and them publication.

### Preoperative planning

**Fig. 1:**
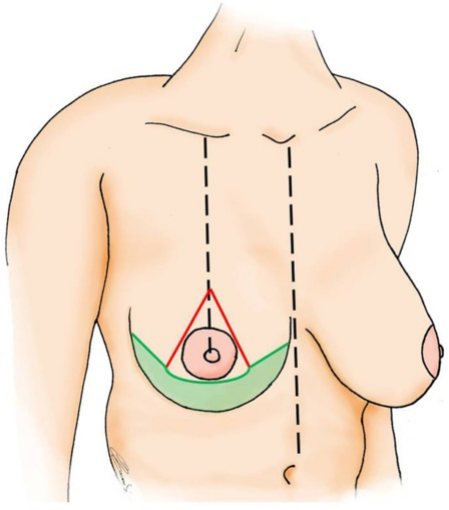
Preoperative planning

The midline was marked with the patient in an upright position. The breast meridian was bilaterally delineated and the future nipple-areola complex (NAC) was determined (19–23 cm from the sternal notch). A slightly modified Wise pattern was then drawn; 2 oblique lines measuring 7 cm were drawn to form an angle of 30° to 90°, according to the amount of excess skin. The other ends of the 2 oblique lines were then extended laterally and medially to join the inframammary fold, as in breast reduction or mastopexy. The area enclosed in the pattern below the NAC was designated the dermal flap (colored in green in Fig. 1).

### Surgical procedure

An appropriate areolotome was used to mark the NAC, which was then excised and stored as a free graft in physiologic saline solution. A frozen section was taken for histological examination to determine if there were malignant cells in the nipple ducts.

**Fig. 2:**
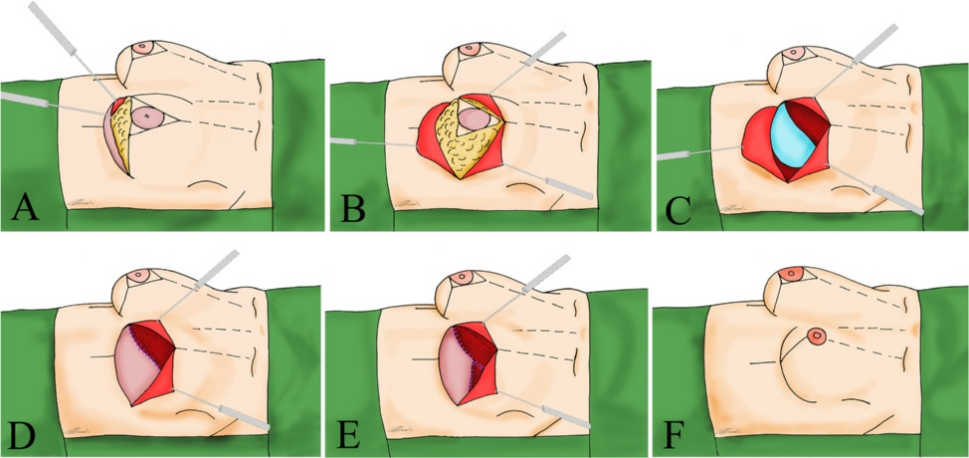
**a** Inferior pedicle dermal flap raising; **b** subcutaneous mastectomy; **c** Implant positioning; **d** Prosthesis coverage suturing dermal flap to inferior border of pectoralis muscle; **e** Serratus muscle elevation to close the pocket lateral aspect, **f** Final sutures

A full-thickness skin incision was made along the 2 oblique lateral lines. The epidermis was incised at the level of the inframammary fold, the area below the NAC and over the inframammary fold was de-epithelialized, and a 1-cm-thick dermal flap was created [Fig. 2a]

Mastectomy was then carried out, with dissection along the subcutaneous plane; Cooper’s ligaments were divided, so that all breast tissue could be removed and the subdermal vascular network could be protected. When indicated, axillary surgery was performed through the same incision [Fig. 2b].

The lateral border of the pectoralis major was identified and elevated to create the pouch. The inferior insertions of the pectoralis major muscle were divided. The free edge of the pectoralis muscle was then sutured to the upper edge of the dermal flap, starting medially. When the suturing was almost completed, an appropriate silicone implant was inserted, followed by completion of the suturing Fig. 2c-d]. The medial aspect of the pouch was generally closed using the dermal flap; in patients where that closure was difficult to perform or traction deformed the pouch profile, a flap of serratus muscle was elevated [Fig. 2e] In selected cases, an acellular dermal matrix (ADM) was positioned to increase the width of the pouch. The skin envelope was then shaped according to the procedure for aesthetic mammoplasty. Care was taken when suturing the skin to reduce tension on the wound. The NAC was then grafted at the appropriate location [Fig. 2f].

### Evaluation of postoperative results

All data were collected retrospectively from the operating theater registers and the surgeons’ logs.

Photographs of the patients were collected before surgery and during follow-up postoperative visits at 2, 6, and 12 months, and used to evaluate the cosmetic results and to determine the outcomes. The photographs consisted of frontal side, lateral, and three-quarter side views.

The photographs were assessed for the following parameters and were graded by 3 external surgeons, and the mean scores were determined:

- Projection of the lower pole related to the body (0 = insufficient - breast mount is not appreciable; 1 = moderate - breast lower pole is defined but without ptosis; 2 = good - lower pole is well defined and ptosis is appreciable; 3 = optimal - lower pole and IMF are well defined with an appropriate ptosis).
- Grade of symmetry (0 = insufficient, 1 = acceptable - breasts present the “physiologic” asymmetry; 2 = optimal - breasts look quite similar)
- Quality of scar and vitality of NAC (0 = insufficient - scars are hypertrophic, visible; second intention healing area are present and the NAC is lost; 1 = acceptable - NAC without projection with some dyschromic areas, scars are quite hypertrophic but hided from the breast; 2 = good - Nipple with light projection, areola well healed scars normotrophic; 3 = optimal - NAC maintains its original aspect, scars are less visible)
- Visibility of prosthesis (0 = always; 1 = in some positions; 2 = never)

The results were estimated from the photographs and data overlapping using a computer graphic program (Anthology - DEKA me.la S.r.l., Calenzano-Florence. Italy) following our outcome scale and then classified as excellent, good, or poor, based on the total score of the parameters (poor = 0–3, good = 4–7, excellent = 8–10).

Patient satisfaction was determined using the modified questionnaire from the Michigan Breast Reconstruction Outcome Scale (Table 2) [2, 17].

**Table 2:** Parameter used to investigate patients satisfaction

1)	Knowing what I know today, I would definitely choose to have breast reconstruction.
2)	Knowing what I know today, I would definitely choose to have the type of breast reconstruction I had.
3)	Overall I Am satisfied with my reconstruction.
4)	I would recommend the type of reconstructive procedure I had to a friend.
5)	I felt that I received sufficient information about my reconstructive options to make an informed choice.
6)	The size and shape of my breasts are the same.
7)	My reconstructed breast feel soft to the touch.

### Statistical analysis

Complications incidence was analysed to find factors related to their onset. Aspects surgery relate such us axillary clearance or ADM implantation have been evaluated. Patient factors like smoking status, diabetes, age and BMI were also studied. The impact of oncologic therapies was studied as well. The Fisher exact test was used to compare the frequency of categorical variables. *t*-test was used to determine statistical significance for categorical data.

The correlation between patient satisfaction score and implant volume was analysed as well. The Pearson correlation test were used to investigate that connection.

Analysis was carried out with SPSS version 22 (IBM Corporation, USA).

## Results

A total of 88 skin-reducing mastectomies were performed (14 patients underwent bilateral procedures). The minimum duration of follow-up was 12 months (mean, 18 months), and no local recurrences were noted but among them 9 have developed systemic disease.

**Fig. 3:**
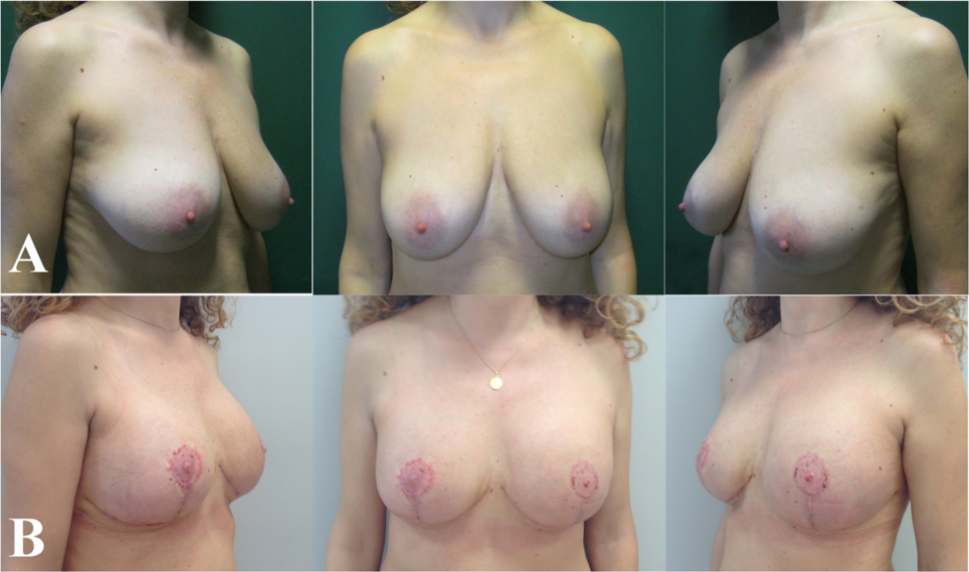
45 years old woman with huge and pendulous breasts, the cancer was located in the right one. **a** Before surgery; **b** 2 months follow-up

**Fig. 4:**
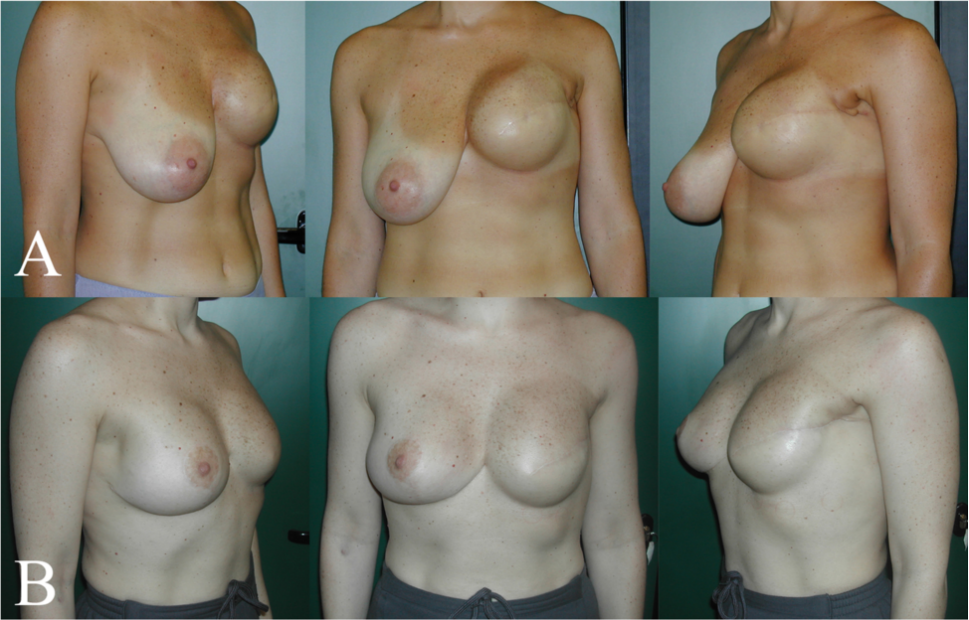
43 years old woman with 3th degree of ptosis on the right breast and previous simple mastectomy and expander implantation on the left. Due the previous multifocal cancer we decided to perform a preventive SRM on the right. **a** Before surgery; **b** 12 months follow-up

**Fig. 5:**
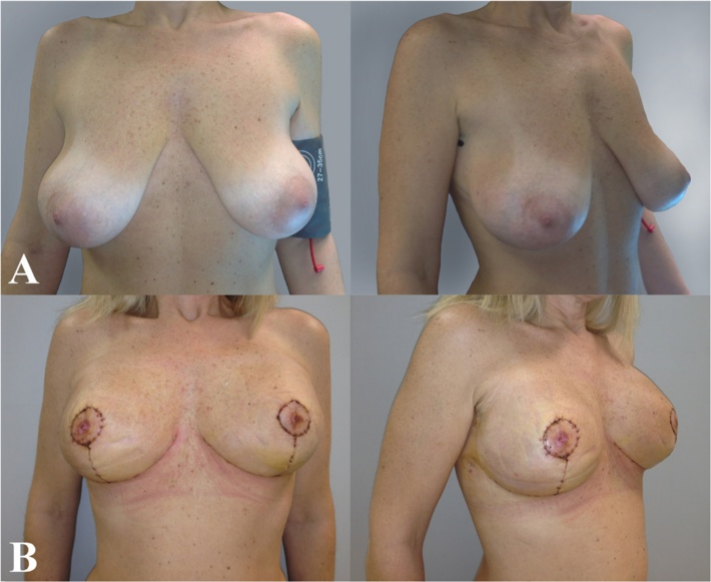
42 years old woman with huge and pendulous breasts, the cancer was located in the right one. **a** Before surgery; **b** 40 days follow-up

Interpolation of the clinical data with our outcome scale led to the following results: excellent for 39 patients (52.7 %), good for 27 (36.5 %), and poor for 8 (10.8 %) [Figs. 3, 4, 5].

Patient satisfaction was as follows: 32 patients (43.3 %) were highly satisfied (score, 7–16), 34 (45.9 %) were moderately satisfied (score, 17–25), 8 (10.8 %) were not satisfied (score, 26–35).

### Oncologic therapy

Because of tumor size and histologic features, 8 patients received neoadjuvant chemotherapy, and 3 months after therapy underwent to skin reducing mastectomy with axillary clearance.

There were 27 patients with metastasis-positive of sentinel lymph node detected at surgery, who underwent axillary lymphadenectomy through the mastectomy surgical incision. These patients received adjuvant chemotherapy after their wound had healed [Table 3].

**Table 3:** Oncological Outcome

Cancer Histology	N of cancers	Lymph node status		
Ductal Cancer	42 cancer (5 patient showed bilateral disease)	35 axillary clearances due to		
Lobular Cancer	12 cancer (6 patient showed bilateral disease)	27 sentinel nodes were positive		8 Patients received neoadjuvant chemotherapy
Cancer stage	N of cancers	N of nodes	cases	
Tis	6	1	5	
T1	27	1 – 4	24	
T2	26	>4	6	
T3	8			
Prophylactic procedures	14 patients (3 bilateral BRCA; 11 previous contralateral cancer)			

Among them 6 patients had indication to post operative radiotherapy.

Fourteen patients received bilateral procedures: 3 were carrier of BRCA mutation and 11 showed bilateral cancer (eight woman had had different cancer histology between the two breasts) 21 prophylactic procedures were performed: 15 patients received unilateral skin reducing mastectomy: 11 for previous contralateral tumor and 4 were positive for *BRCA* genes mutation.

### Nipple-areola-complex reconstruction

The NAC was immediately grafted for 82 breasts; 6 NACs were positive for cancer and therefore not used.

There were 53 (64.4 %) breasts with complete attachment of the NAC grafts; 24 nipples (29.5 %) showed loss of projection, small areas of dyschromia, or small areas of necrosis; and 5 (6.1 %) breasts (6.1 %) lost the NAC. No pedicle NAC flap were created to correct breast ptosis.

Each NAC had been routinely repositioned as a free graft in order to increase the chances for survival. We achieved a high survival rate. Nineteen nipples showed dyschromic areas, 5 patients developed total necrosis of the NAC, and 5 developed partial loss healed by second intentions.

Nipple reconstruction was performed for 4 patients using our previously described technique [18] followed by tattooing the areola, for 4 patients using a tissue graft from the genitalia, and for 3 patients using a star flap.

### Breast implants, ADM patch, and contralateral reshaping

Anatomically shaped silicone gel implants were used (Allergan, Irvine, CA; Mentor, Berkshire, UK). Characteristics of the prostheses are summarized in Table 4.

**Table 4:** Prosthetic characteristic

Average volume (range)	478.6 g (375–750)
Average High (range)	12.84 cm (11.8 – 15.5)
Average Width (range)	14.64 cm (11.8 – 15)
Average projection (range)	5.97 cm (5.3 – 6.9)

For 18 procedures, a serratus muscle flap was used to cover the lateral aspect of the implant. A total of 14 ADM patches were implanted in 10 patients (4 patients received bilateral ADMs).

Contralateral adjustments were performed for 45 patients as follows: 4 women received superior pedicle mammoplasty, 16 patients underwent inferior pedicle reduction mammoplasty, 21 underwent superomedial pedicle breast reduction, and 4 patients following the Toreck technique.

### Complications

There were 22 adverse events (25 % of SRM), including some during the early postoperative period (before the fifteenth postoperative day). The adverse events are shown in Table 5 and their statistical interpretation is summarized in Table 6.

**Table 5:** Complication recorded in our series

Wound dehiscence	3
Cutaneous epidermolysis/necrosis	4
Prosthetic exposure	2
NAC necrosis	5
Seroma	5
Infections	3

**Table 6:**
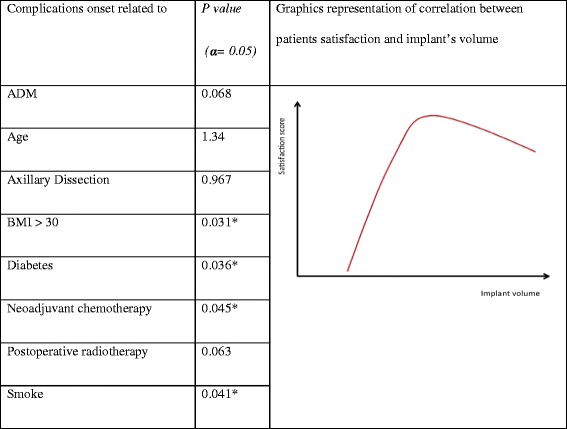
Results statistical analysis

There were 7 complications involving the skin envelope and wound. Superficial epidermolysis near the vertical scar occurred in 4 patients, and minor wound dehiscence occurred in 3 patients. Four of these complications were treated conservatively and 3 patients received a surgical revision. The two patients with exposition of the prosthesis underwent removal of the implant. Six months after implant removal one of these patients underwent a pedicled TRAM flap procedure, which maintains a cutaneous pad to replace the lost skin mound. The second patient refused to undergo autologous reconstruction and 6 months after removal received a tissue expander.

Vascular complications developed mainly in patients who were smokers. There was no statistical difference in the rate of vascular complications in our patients and the rates that have been reported in the literature [8–12].

Implant substitution was performed for 19 breasts (24.5 %), which included 15 performed because of capsular contracture and 4 because patients requested a bigger implant. About 12 months after the initial surgery, fat grafting was performed for 26 patients to improve the coverage of the upper pole of the implant and skin texture.

## Discussion

### Skin reducing mastectomy: history and rationale

Several methods for repairing defects after mastectomies of small breasts have been reported, including autologous tissue transfer [19–22], one- or two-step prosthetic implantation [14, 22, 23], or a combination of the autologous flaps and prosthetic implantation. The main difficulty in performing immediate breast reconstruction using a subpectoral implant is inadequate space at the lower and medial portions of the pouch due to the attachments of the pectoralis major muscle to the ribs [11]. The distal insertions of the muscle are therefore usually divided, placing the implant in the subcutaneous plane.

In women with macromastia, a type IV SSM is often required to achieve an aesthetically pleasing reconstruction. A periareolar approach does not allow an adequate cutaneous excision, and several Wise pattern excisions have been developed to solve the problem [2, 21, 24]. However, these procedures have resulted in several complications that were associated with cutaneous flap skeletonization and inadequate space for placing an appropriate permanent implant [25].

The introduction of the Bostwick technique [15], today known as “skin-reducing mastectomy” [11], which inserts a permanent prosthetic device into a large pouch made by suturing the pectoralis major muscle to the superior border of an inferior pedicle dermal flap [11], allows the surgeon to achieve a safe oncologic result plus a cosmetically satisfying reconstruction. Recentely Querci della Rovere subsequently reported his experience using a two-stage procedure [13].

The inferior pedicle dermal flap enables the construction of a large pouch and acts as a shield between the prosthesis and the skin wound. We believe that this shield is a major advantage of this procedure.

The subcutaneous mastectomy is carried out in a superficial plane immediately under the dermis, and leads to reduced perfusion of the cutaneous envelope, resulting in increased numbers of vascular complications, especially at the T-junction, where wound dehiscence has often been observed. The integrity of the dermal flap prevents direct exposure of the prosthesis to the external environment, reducing the risk of postoperative contamination [11].

In our opinion, an important advantage of this flap is its plasticity, which allows complete coverage of the lower pole of the implant, keeps a well definition of the inframammary fold and provides a soft cushion for the implant.

### Skin-reducing mastectomy and ADM

In patients needing a very large implant, for which the dermal and anterior serratus flaps are inadequate, we use an ADM patch. This biologic material not only provides initial structural strength and bulk, but also allows relatively rapid vascular ingrowth and serves as a scaffold for new tissue formation [26, 27].

A total of 14 ADM patches were implanted in 10 patients (4 patients received bilateral ADMs) and all received ADMs derived from fetal bovine dermis (SurgiMend PRS, Vedise Hospital, Italy).

**Fig. 6:**
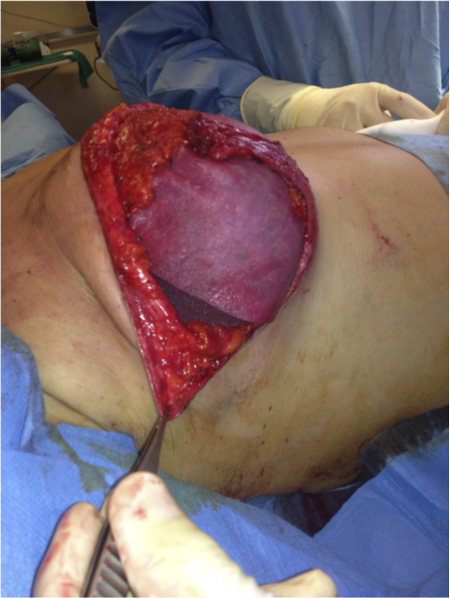
Intraoperative view of ADM implantation, one patch was sutured between the upper margin of dermal flap and the inferior border of pectoralis muscle

Generally, we reserve the use of an ADM for patients who have undergone previous breast surgery such as quadrantectomy, for patients with extremely high insertions of their pectoralis muscle, and for patients needing a large breast implant or large pouch. We have placed these patches on the lateral side of the pouch, thereby avoiding the need to detach the serratus muscle. There were only 2 patients for whom we inserted the ADM between the dermal flap and the free border of the pectoralis muscle, because the distal portion of the flap was not found to have an adequate blood supply [Fig. 6].

In 4 breasts an ADM was almost used as a substitute for the dermal flap, because the tumor was adherent to the skin. We decided not to remove the entire dermal flap and cover the lower pole of the breast implant using an ADM patch, but preferred to leave a 1- to 2-cm flap to place the ADM at the inframammary fold (IMF). This small flap was placed under the incision of the IMF, to prevent exposition of the ADM if wound dehiscence developed reducing the risk of implants infection.

Skin flap quality is important for surgery using a biologic patch. In reconstructive surgery of the breast, only the side of the ADM in contact with tissue is useful for integration; the other side of the ADM is in contact with the implant. If tissue necrosis develops, the ADM cannot be integrated, and then both devices require removal at the very least. No ADMs were used in our study for patients where there was concern about the condition of the skin.

There were complications involving 6 breasts receiving an implant and ADM patch. The most common complication was a seroma persisting for some weeks after surgery. Three patients with seroma underwent percutaneous drainage and systemic antibiotic therapy. Two patients showed early vascular failure signs of the skin envelope and underwent immediate surgical revision to prevent ADM exposition. One patient developed cellulitis of the cutaneous envelope, manifested as swelling and fever, and she underwent removal of the prosthesis.

To minimize manipulation of the ADM patch, we rehydrated the device directly in its original container. After cleaning the surgical field using chlorhexidine, the surgeon attached the ADM patch to the muscle using absorbable suture. The important issue for ADM is correct placement. It is important for preventing the folding or duplication of the patch, which can increase the risk of seroma and lead to failure of ADM integration.

To further decrease the risk of seroma in patients receiving an ADM patch, the drain was left in situ until the daily amount of aspirate was about 10 to 20 mL (in patients who did not receive an ADM patch, the drain was removed when the daily volume of aspirate was 50 mL).

Our complication rate for all patients who received an ADM implantation (not only those undergoing skin reducing mastectomy) appears to be lower than reported in other studies [28].

We believe that our lower complication rates are a result of the narrow indications that we follow for employing ADM patches and of the meticulous intraoperative manipulations of ADM patches. To our knowledge, there are no published similar studies on the complication rate of ADM patches that can be used for specific comparison to our results.

### Comment to results of statistical analysis

We recorded a statistical correlation among complication onsets and some patient life style conditions [Table 6]. BMI and diabetes present the higher level of significance. Flap thickness seems to be the only surgery related factor that increases the risk of complication development. About the ADM use we didn’t record significative relation. Nonetheless we observed high percentage of complication in those patients who received ADM like seroma formation and long lasting drains but this finding it’d be dependent to the small number of patients.

Although our series is smaller, we concord with Korwar study didn’t find a significative correlation of SRM with postoperative radiotherapy, nevertheless the 6 patient who received ionizing therapy request more adjuvant procedure to maintain the result such us lipofilling or implant substitution after capsular contraction [29].

The most important find is highlighted by Pearson correlation test that showed an atypical not linear relation [Table 6]. In fact we observed a linear correlation between satisfaction score and implant volume up to 475 cc then a trend inversion was recorded. We went to study this phenomenon looking for patients who received wider implant and reported lower score. We discover that they had BMI higher the 31. This finding is well known in literature where is highly reported obesity have to be consider as exclusion criteria to implant based breast reconstruction.

### Tips and tricks and some possible variations

During creation of the dermal flap, care must be taken to preserve the integrity of the inframammary fold to allow a natural-appearing and physiologic ptosis [30]. We do not agree with Prathap [31], who recommends suturing the dermal pedicle to the thoracic wall to better define the inframammary fold, because that procedure compromises vascularization, leading over time to reduction in the volume of the lower pole. We also do not agree with Hammond [16] and Querci della Rovere [13] to place an expander, because in patients where survival of the skin envelope is doubtful or a two-step reconstruction is scheduled, we prefer to perform a traditional mastectomy followed by placement of a breast expander into a muscular pouch.

It is our opinion that skin is placed at risk not when the prosthesis presence generate high tension on cutaneous surface but when there is vascular damage after a poorly performed mastectomy, carried out too much superficial exposing the dermis.

Indeed, for 2 of our study patients who had skin necrosis in the area of the lower pole, the viability of the dermal flap resulted in second-intention healing.

Regarding patients with an insufficiently large dermal flap, we do not agree with Colizzi et al., who recommend increasing the mobility of the flap by detaching the lateral insertion of the flap along the inframammary fold, because this technique will reduce the vascular support of the flap [32]. We prefer covering the lateral aspect of the device, creating a flap using the serratus, which we performed for 18 study patients.

We would like to emphasize again that it is very important when creating the flaps to perform a very careful subcutaneous dissection to preserve the integrity of the vascular network. Indeed, we prefer to perform the mastectomy using a blunt scissors to avoid thermal injury to the skin flap caused by electrical devices. We have found that careful dissection aids in maintaining the integrity of the dermal and subcutaneous vessels, thereby increasing the survival rate of dermal flaps.

## Conclusions

Nava et al. reported that the skin-reducing mastectomy allows the surgeon to perform immediate breast reconstruction and achieve a satisfactory outcome. According to our outcome scores, we achieved excellent results for the majority of our patients; however, as for other breast reconstructions that use prosthetics, surgical revision may be needed to maintain the results quality.

There were some discordant results for patient satisfaction. Although most of the patients with optimal clinical results had high scores on the questionnaire from the Michigan Breast Reconstruction Outcome Scale, indicating a high degree of satisfaction; some patients, despite good clinical results, reported low personal satisfaction. This finding may reflect the extremely high expectations of some patients who undergo breast reconstruction. Some of these patients may not understand that a breast reconstruction is not a cosmetic mammoplasty. We observed this finding more commonly in patients who underwent to immediate breast reconstruction instead of a multistage procedure.

The long-term outcomes of skin-reducing mastectomy have not been clarified. The long-term followup of some of our study patients has revealed progressive distortion of the breast profile associated with weight changes of the patient and/or capsular contracture. We performed implant substitution for 19 breasts (21.5 %), 15 for capsular contracture, and 4 patients requested a bigger implant. Capsular contracture was observed more frequently in patients with thin dermal flaps or with very large implants. It is well known that the principal cause of capsular contracture is inadequate coverage of the implant.

Another important finding was the visibility of the upper pole of the implant after complete tissue healing. This has been seen with other SSM procedures. The upper edge of the implant is often visible because of skin adhesions to the muscle. To improve coverage of the upper pole of the implant, fat grafting was performed for 26 patients approximately 12 months after SRM. The great part of this patient received a full high prosthesis. In creating the prosthetic pocket, we occasionally elevated a flap of serratus muscle. However, this procedure is only used in cases of excessive lateral skin flap skeletonization or if the insertions of the pectoralis muscle are too high.

The use of ADM patches in skin-reducing mastectomies remains controversial, because the risk of, local complications should be higher than for traditional surgical procedures.

Is it preferable to place a breast implant laterally into the subcutaneous plane than expose the patient to increased risk of complications? There are many disagreements regarding this question. We think that use of the ADM patch should be confined to experienced surgeons, who can provide the expertise to allow this scaffold to be used safely. Therefore, we prefer to implant a biologic patch than perform a simple mastectomy. To prevent exposition of the ADM patch, we have found that it is important to place the biologic patch at a distance from surgical incisions. Therefore, if the dermal flap must to be removed because of oncologic indications, we always preserve at least 15 to 20 mm of dermal flap in order to fix the biologic patch above the incision of the inframammary fold. The placement of an ADM between the pectoralis muscle and dermal flap enables increased projection of the new breast as well as a wide pouch.

In cases of jeopardize skin we suggest performing an immediate surgical revision in order to preserve the implants and prevent their exposition and contamination.

We believe that skin-reducing mastectomy with immediate placement of a permanent prosthesis is an indispensable procedure for the treatment of women with breast cancer in a large and pendulous breast. However, determining if the patient is suitable for the procedure is extremely important, because the risks of skin necrosis, implant infection, and subsequent removal are not negligible.

Skin-reducing mastectomies performed by skilled surgeons achieve good results and patient satisfaction; however, as for other breast-conserving surgeries, for some patients it may not be the ultimate solution, and may require surgical revision to obtain optimal results.
